# Batteryless BLE Module with a Piezoelectric Element Mounted on a Shoe Sole

**DOI:** 10.3390/s24092829

**Published:** 2024-04-29

**Authors:** Shusei Dan, Yusuke Yano, Jianqing Wang

**Affiliations:** Graduate School of Engineering, Nagoya Institute of Technology, Nagoya 466-8555, Japan; s.dan.631@nitech.jp (S.D.); yano@nitech.ac.jp (Y.Y.)

**Keywords:** piezoelectric element, BLE, energy harvest

## Abstract

A position identification system for wandering elderly people uses BLE to transmit ID information. The objective of this study is to make the BLE module batteryless using a piezoelectric element. The piezoelectric element is mounted on the sole of a shoe, and when pressure is applied to the piezoelectric element by walking, a voltage is generated between both electrodes of the piezoelectric element. This voltage is used to store the necessary power as a battery to operate the BLE module. In this paper, we provide a step-by-step design approach using piezoelectric elements attached to a shoe to power an actual BLE module. We derive an equivalent circuit for the piezoelectric element under walking conditions and, through circuit simulation and actual measurements, clarify the amount of time required to charge the voltage to drive the BLE, demonstrating the possibility of a batteryless BLE module for use in locating a wanderer while they are walking.

## 1. Introduction

The elderly are affected by many diseases, including degenerative neurological diseases, vascular disorders, Alzheimer’s disease, and dementia. The symptoms of dementia include reduced comprehension and judgment, increased forgetfulness, and, in some cases, wandering outside the home and not being able to return [1,2]. As a result, elderly people with dementia may be out of sight or out on their own, even within nursing care facilities. It is reported that about 10,000 elderly people with dementia become lost each year in Japan only, and many people also suffer from this same problem in other countries all over the world. As a solution that identifies an elderly wanderer’s position, global navigation satellite system (GNSS) terminals are typically used. However, they require charging every few days. In contrast, Bluetooth low energy (BLE) can operate on a very low consumption of power, and BLE signals can easily be received by smartphone users within dozens of meters [3,4]. Therefore, a system using BLE at a 2.4 GHz band has been developed for elderly wanderer position identification [5,6]. The system provides an elderly person with a BLE module mounted on a shoe, which transmits personal ID information to smartphones owned by people who happen to pass nearby. By installing software on a smartphone in advance, it can send its position signal to a server after receiving the BLE signal, and then the position of the elderly person can be identified based on the smartphone’s position and his/her family informed.

However, given that the target population is elderly, regular recharging and battery replacement are expected to be difficult. A promising approach is to embed piezoelectric elements in the soles of shoes to supply power to the BLE module when walking. This is because a piezoelectric element is a passive material that converts the force applied to it into a voltage [7]. Over the last few years, piezoelectric elements have emerged as potential and reliable options for harnessing ambient mechanical vibrations to generate usable electrical energy. They have been used to operate sensor networks and wearable devices for personal health monitoring by converting the mechanical vibrations caused by human motion into electrical power [8,9,10,11]. As a typical example, by attaching a piezoelectric element to the sole of a subject’s shoe, a voltage can be generated by walking and supplied to a wearable device without putting a burden on the subject [12,13]. In [14], a practical application of a piezoelectric shoe-based RFID device was demonstrated using various piezoelectric materials, such as polyvinylidene fluoride, and piezoelectric unimorph devices. In [15,16,17], the use of piezoelectric elements was demonstrated for energy harvesting to power GPS devices, IoT devices, or smartphones. The energy-harvesting performances under different conditions, such as walking, jogging, and running, with two rectifier configurations were investigated in [18], and the power generated at different parts of a shoe was investigated in [19]. Moreover, four types of connection configurations for circular disc piezoelectric transducers on sole pads, which are named series, parallel, series–parallel, and parallel–series topologies, were designed and tested in [20]. All of these studies focus on demonstrating the feasibility of attaching piezoelectric elements to shoes to generate power and drive a low-power device. However, for any application of piezoelectric energy harvesting, an appropriate interface circuit is required to serve its purpose [21,22]. When designing an interface circuit, an equivalent circuit of a piezoelectric element with the appropriate internal electrical impedance is a determining factor in optimizing the power harvesting. An appropriate estimation of the load impedance of the device to be powered is also an important factor. However, the abovementioned studies lack a detailed approach that shows how to derive an equivalent circuit of the piezoelectric element and how to derive the load impedance of the device to be powered in order to design an appropriate interface circuit to convert the pressure on the shoe into a voltage.

In this study, we aimed to develop a batteryless BLE module using piezoelectric elements attached to the sole of a shoe. Compared to the abovementioned literature, this paper presents (1) a method of constructing equivalent circuits for real piezoelectric elements based on measurements, (2) a method of checking whether the parameters of the equivalent circuit change with the pressure and weight of the piezoelectric elements used, and (3) a circuit design method of powering an actual BLE module based on its characteristics shown in data sheets. The novelty of this paper is that it focuses on providing a step-by-step design approach for a real BLE module.

**Figure 1:**
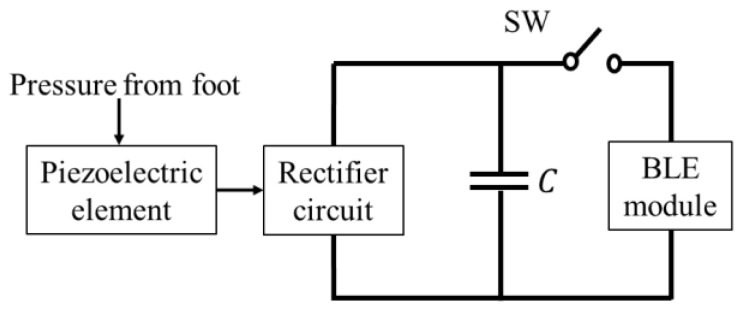
Block diagram of the system.

The operation of a BLE module requires a DC voltage, but the DC voltage cannot be directly obtained from the piezoelectric element due to the pressure applied to the foot when walking. In other words, the piezoelectric element alone cannot operate the BLE module. As shown in Figure 1, the voltage obtained from the piezoelectric element is first charged to a capacitor by a rectifier circuit to generate the DC voltage that drives the BLE. The switch (SW) between the BLE module and the capacitor for the power supply is turned off at this time. The charging time should be a few minutes for practical use. The charged power supply capacitor is connected to the BLE module by turning on the SW. The BLE module is then activated and transmits at least one packet of position information.

To develop an approach to deriving an appropriate interface circuit for power supply using piezoelectric elements, first, we calculate the minimum capacitance value of the capacitor for the BLE’s power supply and the charge voltage value required for the BLE to transmit one packet of position information. Second, we develop an equivalent circuit for the piezoelectric elements and the rectifier circuit that can generate the required voltage under the calculated capacitance of the power supply capacitor by simulation and measurements. Third, we experimentally validate the above approach, and, finally, we use the developed circuit for a real BLE module to demonstrate the usefulness of the approach.

The organization of this paper follows the design process of a circuit that uses piezoelectric elements to supply power to an actual commercially available BLE module. Section 2 describes how to determine the capacitance and voltage values required for a BLE power supply capacitor. Section 3 describes the detailed derivation of the equivalent circuit for a piezoelectric element. Section 4 described an experimental verification of the charging voltage using the circuit design based on the derived equivalent circuit of the piezoelectric element, and Section 5 describes a verification experiment in which a batteryless BLE module with piezoelectric element mounted on the shoe sole is actually used to transmit ID information for demonstrating the usefulness of this design approach. Section 6 concludes this study.

## 2. Required Capacitance and Voltage Values for BLE Power Supply Capacitor

On the basis of the BLE module specifications, we calculated the capacitance value of the power supply capacitor and the charging voltage value for the capacitor required for the power-on reset (POR), initialization processes, and transmission of one packet. When the SW in the circuit, as shown in Figure 1, is turned on, the BLE module enters the active mode after the time required for the POR and initialization processes from the shutoff state has elapsed, and the information packet is transmitted after this initialization. In this paper, the POR and initialization processes are collectively defined as initialization.

**Figure 2:**
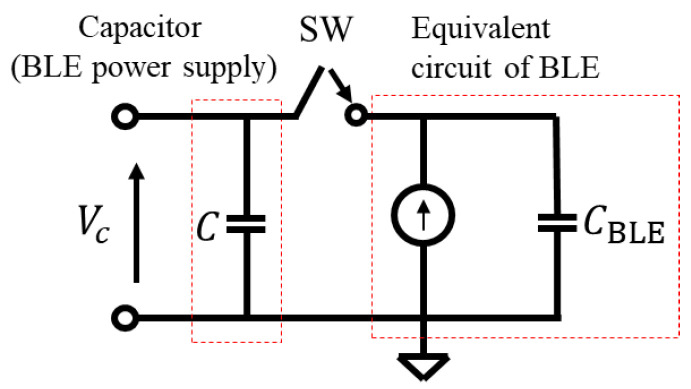
Equivalent circuit for the BLE module when the BLE transmits a packet.

**Figure 3:**
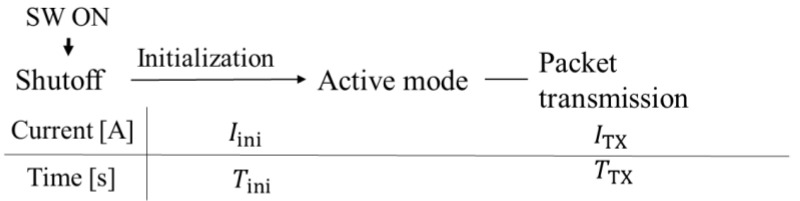
Flow of the BLE module’s operation.

The equivalent circuit of the BLE IC during transmission can consist of a parallel circuit of a current source and an on-module decoupling capacitor, $C_{BLE}$, as shown in Figure 2. The current source represents the DC current defined as the average current consumption during the BLE module’s operation ($I_{ini}$ for initialization and $I_{TX}$ for packet transmission) within its operating voltage range ($V_{min}$–$V_{max}$). Figure 3 shows the flow of the BLE module’s operation, where $T_{TX}$ is the time required to send one packet, and $T_{ini}$ is the time required for initialization. Let $Q_{ini}$ be the total charge required for initialization; $Q_{ini}$ can be obtained as $Q_{ini}=I_{ini}T_{ini}$. Let $V_{max}$ be the maximum possible operating voltage and $V_{min}$ be the minimum possible operating voltage. Equation (1) can be used to calculate the minimum capacitance of the capacitor that can supply DC voltage to transmit a single packet. Since the charge required for the BLE module’s operation is supplied only from the power supply capacitor, C, the voltage at C decreases with time as the BLE module starts to operate, but the voltage supplied to the BLE module needs to be maintained between $V_{min}$ and $V_{max}$ during the period of time from initialization to the transmission of one packet. In other words, the BLE module can be operated with the minimum amount of charge by determining the capacitance of C so that the voltage of C before the BLE module starts operating is $V_{max}$ and that of C after one packet is sent is $V_{min}$. The charging time by the piezoelectric element needs to be minimized. When the SW is turned ON from OFF, the charge of the power supply capacitor, C, moves to $C_{BLE}$, and $V_{c}$ becomes small. To ensure the power supply voltage is sufficient to maintain the BLE module during packet transmission, the charge voltage to the power supply capacitor (C), $V_{c}$, should satisfy Equation (2).

(1) $$\begin{array}{c} C=\frac{I_{ini}T_{ini}\;+\;I_{TX}T_{TX}}{V_{max}\;-\;V_{min}} \end{array}$$

(2) $$\begin{array}{c} V_{C}=\frac{C\;+\;C_{BLE}}{C}V_{max} \end{array}$$

Most of the values of the parameters in Equations (1) and (2) can be obtained from manufacturer’s data sheets. As an example, a BLE module of Infineon (CYBLE-02001-00) [23] was used in this study. The BLE module specifications are as follows: I_wake_ = 1.7 mA, I_Tx_ = 16.5 mA, T_wake_ = 2 ms, V_min_ = 1.7 V, and V_max_ = 5.5 V. For $C_{BLE}$, referring to the data sheet, it was calculated from the composite capacitance between the supply voltage terminal and ground as 2.3 µF. Therefore, once $I_{ini}$, $T_{ini}$, $I_{TX}$, $T_{TX}$, $V_{max}$, and $V_{min}$ are known, the minimum required capacitance of the power supply capacitor can be determined from Equation (1) and the minimum required charge voltage from Equation (2). However, for this BLE module, the $I_{ini}$ and $T_{ini}$ are not provided. Instead, the $I_{wake}$ and $T_{wake}$ of the wake up are given. Usually, initialization is a process from the shut-off to active modes, and wake up is a process from the sleep mode to the active mode. So, $T_{wake}$ should be shorter than $T_{ini}$. In this study, since we had no data on $I_{ini}$ and $T_{ini}$ from the data sheet, we adopted $I_{wake}$ and $T_{wake}$ to replace $I_{ini}$ and $T_{ini}$ as a first step. In this case, the calculated minimum required capacitance of the power supply capacitor is 368.6 µF for the data rate of 10 kbps and 5.8 µF for the data rate of 1 Mbps.

In addition, the capacitance of C can be further confirmed by actual measurement. First, a variable capacitor with the capacitance determined according to Equation (1) is charged to 3.8 V by a regulated power supply. The capacitor is then connected to the BLE power supply terminal with the SW turned on, and the BLE module is operated by the capacitor’s charged power. The information transmission from the BLE module is confirmed by checking whether it can be received by a smartphone application software (BLE scanner 4.0). Since the capacitance of the power supply capacitor is sufficiently larger than the bypass capacitor of the BLE module, the voltage drops the moment the SW is turned on, but it is small enough and is not taken into account. If the smartphone application receives the information sent by the BLE module, this means that the calculated capacitance of the power supply capacitor is sufficient. If the smartphone application does not receive the information, the capacitance of the power supply capacitor should be increased until the information is received. The capacitance when the smartphone application is able to receive the information is the capacitance of the power supply capacitor. For the BLE module used in this study, this capacitance is determined as 1620 µF based on the above-described method. The value is four times the calculated minimum capacitance of the power supply capacitor when the charging voltage is 3.8 V. This is because we used $I_{wake}$ and $T_{wake}$ instead of $I_{ini}$ and $T_{wake}$ in Equation (1) for the calculation. The total charge required for initialization is considered to be four times the sum of the total charge required for wake up and packet transmission.

## 3. Derivation of Equivalent Circuit of Piezoelectric Element

**Figure 4:**
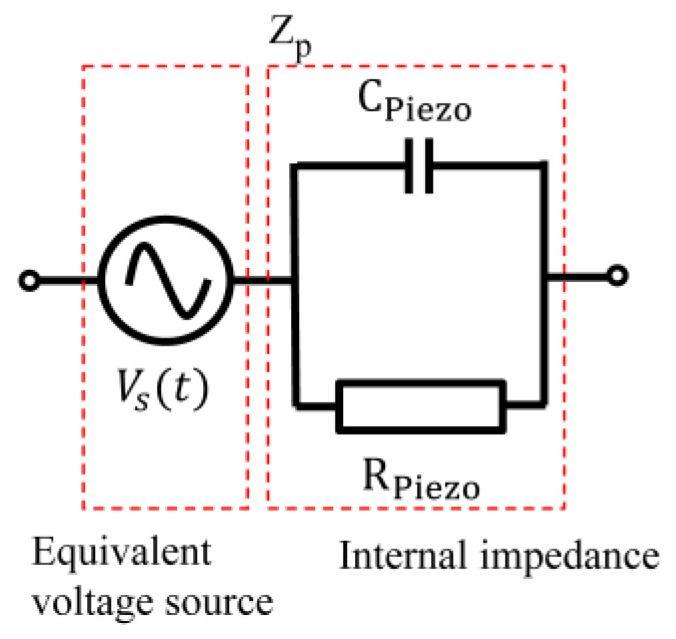
Equivalent circuit of the piezoelectric element.

The equivalent circuit of the piezoelectric element consists of internal impedances, $R_{piezo}$ and $C_{piezo}$, and an equivalent voltage source, $V_{s}(t)$, as shown in Figure 4. In most of the equivalent circuits [22,24,25,26], the current source and internal impedances are represented as a parallel circuit. But in this study, the current source is equivalently converted to a voltage source based on Thevenin’s theorem, and the equivalent circuit can be represented by a voltage source and internal impedance. These parameters are derived to enable circuit simulation and design.

### 3.1. Derivation of Internal Impedance

To derive the internal impedance of a piezoelectric element in its equivalent circuit, the frequency characteristic $\left|Zp(f)\right|$ of the internal impedance of the piezoelectric element was measured with an LCR meter (Hioki, IM3536), and then the frequency characteristic $\left|Zp(f)\right|$ of the equivalent circuit model was approximated by the least-squares method to obtain $R_{piezo}$ and $C_{piezo}$. $\left|Zp(f)\right|$ is expressed as follows:

(3) $$\begin{array}{c} \left|Zp\left(f\right)\right|=\frac{1}{\sqrt{{\left(\frac{1}{R_{piezo}}\right)}^{2}\;+\;{\left(2\pi fC_{piezo}\right)}^{2}}} \end{array}$$

In this study, the frequency range of the impedance measurement was set to 1 Hz–100 kHz. The piezoelectric element was mounted on the sole of a shoe, and pressure was applied by walking. Considering the possibility that the internal impedance changes depending on the applied pressure, we measured the frequency response of the impedance while changing the applied pressure. The applied pressure was assumed to be time-invariant, and since the pressure applied to the foot during walking ranges from 0 N to 700 N [27], the frequency response was measured while forces of 50 N, 100 N, 300 N, 500 N, and 700 N were applied to the piezoelectric element.

**Figure 5:**
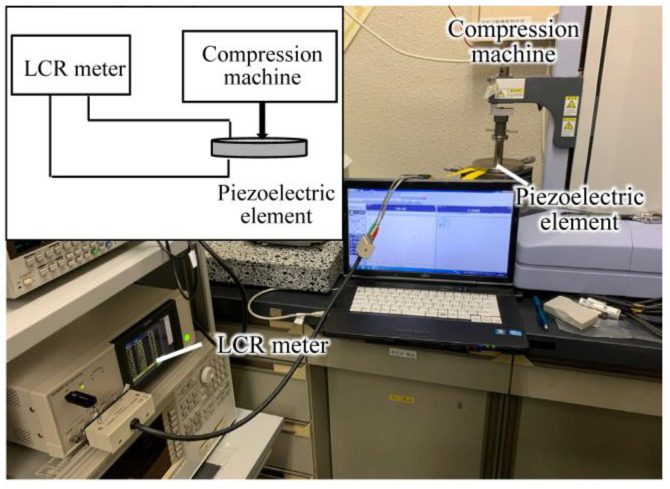
Setup for measuring the impedance frequency characteristics against pressure.

**Figure 6:**
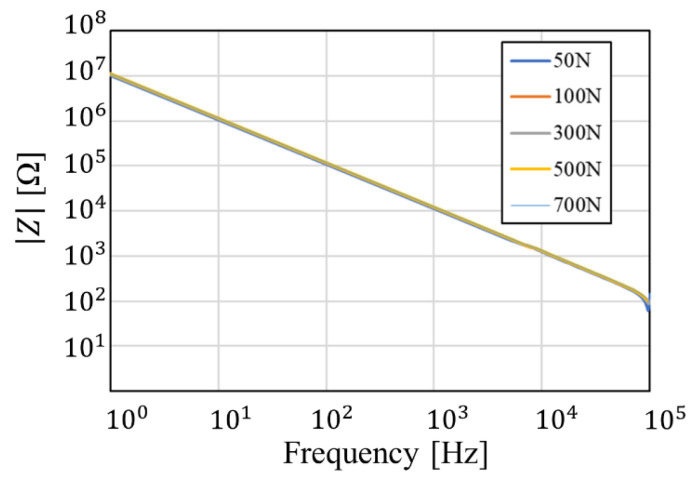
Measured impedance versus frequency under various pressures for piezoelectric element A.

The setup for measuring the impedance frequency characteristics against pressure and the measurement results for piezoelectric element A (Murata, 7BB-27-4) are shown in Figure 5 and Figure 6, respectively. Element A consisted of a metal (i.e., brass) layer 27 mm in diameter, a ceramic layer 19.7 mm in diameter, and a pair of silver electrodes 18.2 mm in diameter. The resonant frequency was 4.6 kHz.

As a result, the internal impedance of the piezoelectric element did not change with pressure. Therefore, the internal impedance in the equivalent circuit of piezoelectric elements is assumed to be unchanged with pressure, and piezoelectric element A has $R_{piezo}=37.37$ MΩ and $C_{piezo}=18.27$ nF. In addition, no change due to pressure was confirmed for another piezoelectric element B (Murata, 7BB-12-9), and the values were R_piezo_ = 81.67 MΩ and $C_{piezo}=8.84$ nF. The main differences between the two elements are size and resonant frequency. Element B consisted of a metal (i.e., brass) layer 12 mm in diameter, a ceramic layer 9 mm in diameter, and a pair of silver electrodes 8 mm in diameter. The resonant frequency was 9.0 kHz. In the subsequent studies, we adopted the piezoelectric element A because it has higher power conversion efficiency to pressure.

### 3.2. Derivation of Equivalent Voltage Source

**Figure 7:**
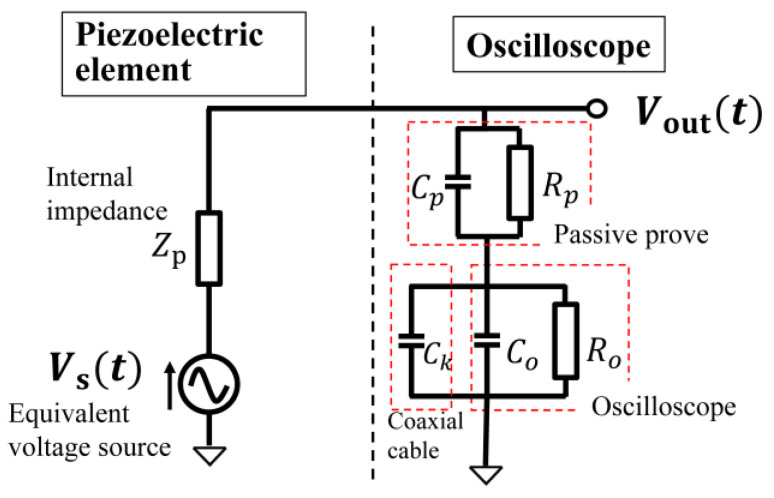
Equivalent circuit diagram of the measurement system.

**Figure 8:**
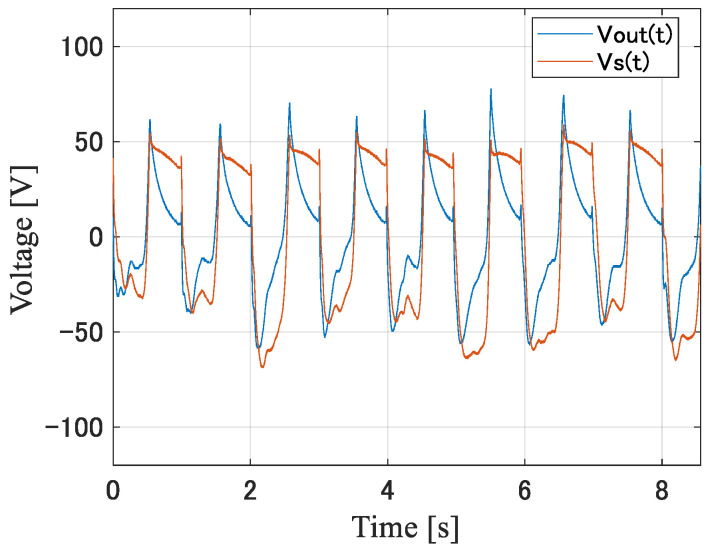
Measured $V_{out}(t)$ and derived $V_{s}(t)$ for element A.

This section describes the derivation of an equivalent voltage source in the equivalent circuit of the piezoelectric element. First, a piezoelectric element was attached to the sole of a shoe. Then, the voltage $V_{out}(t)$ generated by the piezoelectric element while walking with both feet at a pace of 2 steps per 1 s was measured with an oscilloscope. Let the equivalent voltage source be $V_{s}(t)$, considering the oscilloscope (Teledyne LeCroy, WAVEACE2024) and passive probe (Tektronix, P6139A) used in the measurement, and the equivalent circuit diagram of the measurement system is shown in Figure 7. $C_{p}$ and $R_{p}$ are the capacitance and resistance of the passive probe, $C_{o}$ and $R_{o}$ are the capacitance and resistance of the oscilloscope, and $C_{k}$ is the capacitance of the coaxial cable of the passive probe. Their values are as follows: $R_{p}$ = 9 MΩ, $R_{o}\;$= 1 MΩ, $C_{p}\;$= 12 pF, $C_{o}\;$= 18 pF, and $C_{k}\;$= 100 pF. The measured voltage waveform, $V_{out}(t)$, at piezoelectric element A and the time waveform of the piezoelectric element equivalent voltage source, $V_{s}\left(t\right)$, derived based on a circuit analysis of Figure 7, are shown in Figure 8. Note that vs. is the equivalent voltage source when walking, and *V_out_* is the voltage observed in the oscilloscope. In Figure 7, the impedance seen from the piezo element to the oscilloscope is the load of the equivalent circuit of the piezo element consisting of the equivalent voltage source, *V_s_*, and the internal impedance. So, vs. is divided by the internal impedance and the load, and the voltage at that load is *V_out_*. The equivalent voltage source, *V_s_*, shows a change in the generated voltage due to the variation in pressure over time.

## 4. Measurement of Charging Voltage

**Figure 9:**
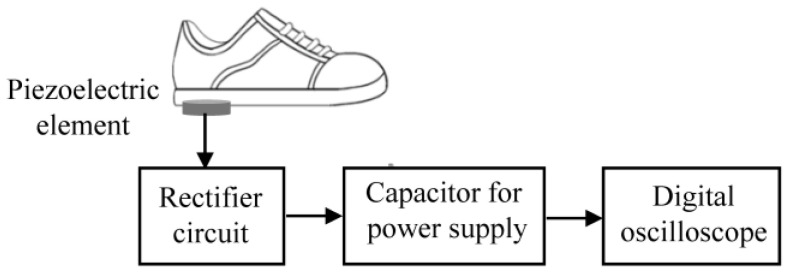
Block diagram for the charging voltage measurement.

**Figure 10:**
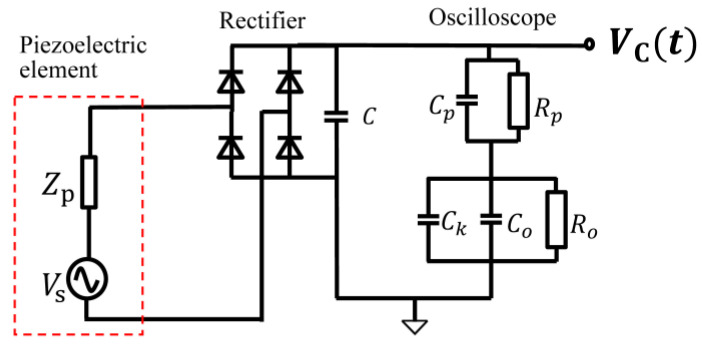
Circuit in the SPICE simulation.

The time required to reach a charging voltage for the power supply capacitor was examined by actual measurement using piezoelectric element A, which has higher power conversion efficiency to pressure. The walking pace was the same as in Section 3 (2 steps/s). The experiment setup for the charging voltage is shown in Figure 9. At the same time, circuit simulations were performed using LTspice for the circuit configuration shown in Figure 10 for comparison. The parameters of element A were $R_{piezo}=37.37$ $M\Omega ,C_{piezo}=18.27$ nF, and $V_{s}(t)$ in Figure 8. The capacitances of the power supply capacitor were first chosen to be 400 µF (approximately 368.6 µF derived as a first step in Section 2) and its half of 200 µF, and the target charging voltage was set at 3.8 V. In both experiments and simulations, a rectifier circuit, consisting of four diodes, was used to charge the power supply capacitor. Among the electrical characteristics of the diodes, forward voltage and reverse leakage current are considered to affect the charging voltage and charging time, and two types of diodes (A: PRE07VS4S, B: RR2L4STE25) were used, to which the diode models corresponding to LTspice are applicable. Their electrical characteristics are shown in Table 1. $I_{F}$ is the forward current, and $V_{R}$ is the reverse voltage.

**Figure 11:**
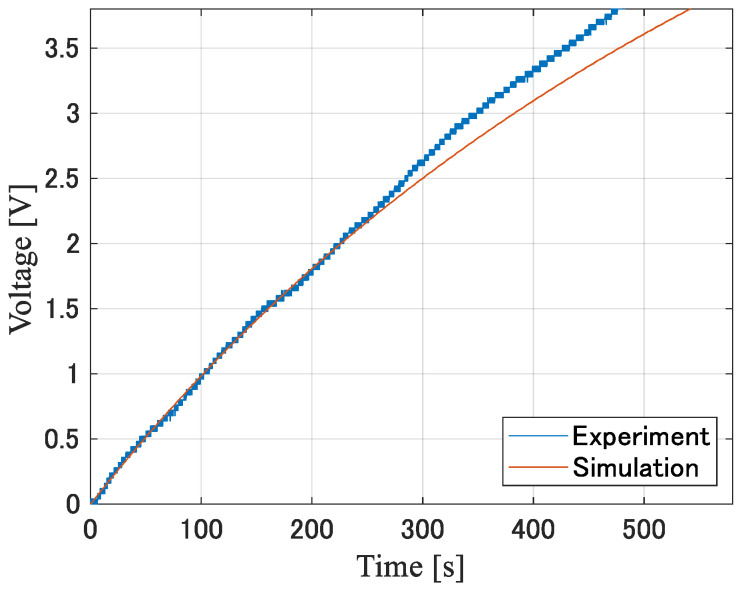
Charging voltage when using diode A for the 400 µF capacitor.

**Figure 12:**
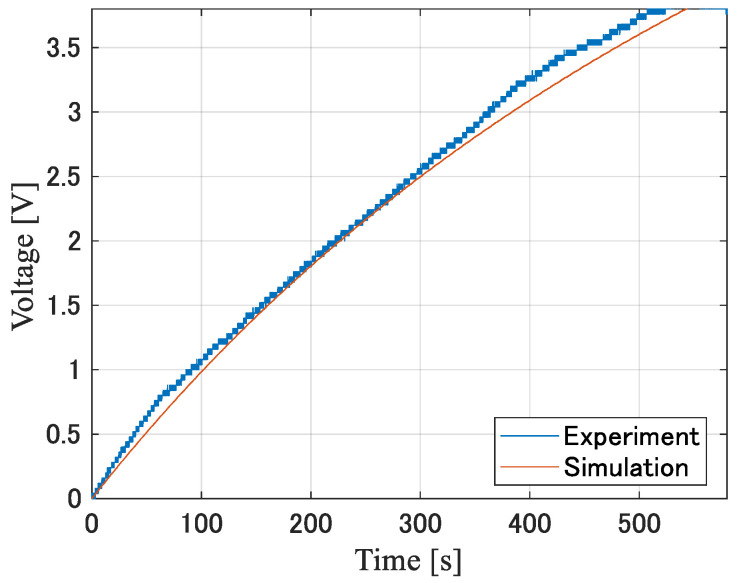
Charging voltage when using diode B for the 400 µF capacitor.

Figure 11 and Figure 12 show the simulated and measured charging voltage time waveforms when using diodes A and B, respectively, when the capacitance of the power supply capacitor is 400 µF. Table 2 shows the time required to boost the charging voltage, $V_{c}$, to 3.8 V obtained from the actual measurement and simulation. In addition, five measurements were taken to account for footstep variations. The average value and standard deviation of the time required to reach the charging voltage for element A are 494.2 s and 48.0 s, respectively. One possible reason for the variation with a standard deviation of 48.0 s is that the pressure applied to the subject’s foot during walking was not constant. We confirmed that the peak value of the voltage, $V_{out}(t)$, induced by the piezoelectric element changed by more than 25 V when the subject stepped a little harder. Therefore, the peak value of the equivalent voltage source, $V_{s}\left(t\right)$, at the piezoelectric element changed to some extent depending on the magnitude of the pressure applied to the piezoelectric element.

## 5. Verification Experiment as the BLE Power Supply

**Figure 13:**
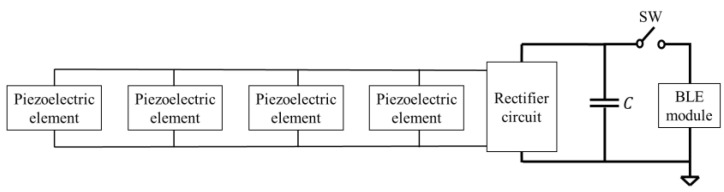
Block diagram when four piezoelectric elements are connected in parallel.

As indicated in Section 2, the capacitance of the power supply capacitor obtained from the measurement is four times the capacitance calculated from Equation (1) by replacing $I_{ini}$ and $T_{ini}$ with $I_{wake}$ and $T_{wake}$, so the charging time is also expected to be four times longer, or about 20 min. Transmitting information once every 20 min is too long for the position identification of a wanderer. A charging time of a few minutes is desirable. To achieve a charging time of a few minutes, the charging time must be reduced to one-quarter. Therefore, the verification experiment for powering the actual BLE module (Infineon, CYBLE-02001-00) was conducted using not only a single piezoelectric element but also four piezoelectric elements. The four piezoelectric elements were stacked, and electrodes were connected in parallel, as shown in Figure 13.

**Figure 14:**
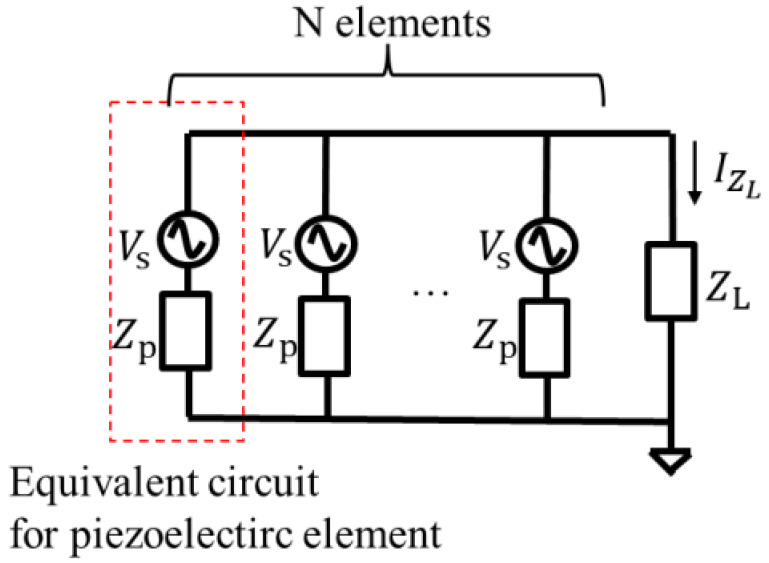
Equivalent circuit of *N* piezoelectric elements connected in parallel.

The equivalent circuit of *N* piezoelectric elements connected in parallel is shown in Figure 14. $Z_{p}$ is the internal impedance of the piezoelectric element. When $\left|\frac{Z_{p}}{N}\right|\gg \left|Z_{L}\right|$ is satisfied, where $Z_{L}$ is the load impedance, the current $I_{Z_{L}}$ flowing into the load $Z_{L}$ is *N* times larger than that with one piezoelectric element when *N* piezoelectric elements are used. Since $Z_{p}$ is about $10^{5}$ times larger than $Z_{L}$ at frequencies of 0–50 Hz, the condition $\left|\frac{Z_{p}}{N}\right|\gg \left|Z_{L}\right|$ is sufficiently satisfied when four piezoelectric elements are used. Therefore, by stacking four piezoelectric elements and connecting their electrodes in parallel, the current flowing in the capacitor, C, for the power supply will be quadruple and the charging time will be reduced to a few minutes.

**Figure 15:**
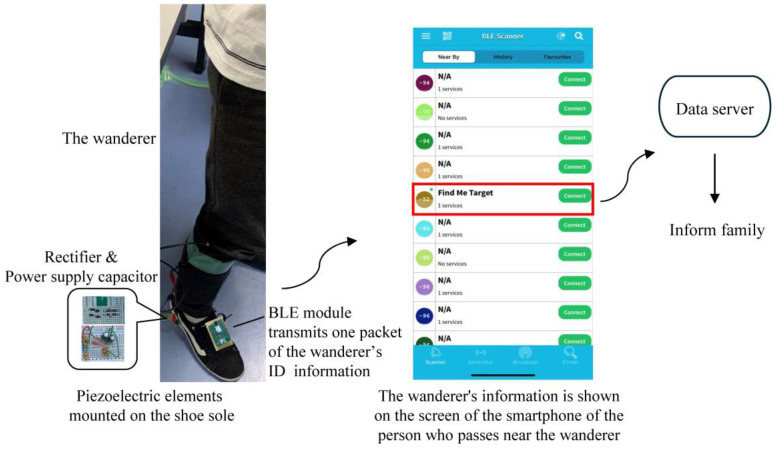
Experiment scene and the screen of a smartphone of a person who passes near the wanderer. The smartphone detects the wanderer based on ID information transmitted from the BLE module and automatically sends his position information to a data server to inform his family.

Same as in previous sections, a piezoelectric element (element A) was attached to the sole of a shoe, and the shoe was stepped on at a pace of 2 steps/s. When the capacitor with a capacitance of 1620 µF or 940 µF for the BLE module power supply reached the target charge voltage of 3.8 V or 5.5 V, the SW between the BLE module and the capacitor was turned on. The capacitor capacitance, charging voltage, and the time required for charging to the desired voltage are shown in Table 3. When four piezoelectric elements (element A) were used, one packet of information was successfully transmitted from the BLE module to the smartphone by turning on the SW after approximately 5 min of charging time by walking. Figure 15 shows the experiment scene and the screen of a smartphone application software that detected a wanderer based on ID information transmitted from the BLE module. This result meets the goal of this study. In such a way, the position of the wanderer can be automatically transmitted to a server by the smartphone, because the smartphone is in the same position as the BLE module and its position is always easily determined by the GNSS. It is also confirmed from Table 3 that the use of four piezoelectric elements reduced the charging time to one-quarter of that of a single element under the conditions of a power supply capacitor of 1620 µF and a charging voltage of 3.8 V. Furthermore, a higher charging voltage of 5.5 V can be used to obtain a shorter charging time of 230 s with a smaller capacitor of 940 µF.

## 6. Conclusions

In this study, aiming at using a powerless BLE module for elderly wanderer position identification, we proposed a design method and developed a power-generating circuit using piezoelectric elements on the sole of a shoe to supply power to the BLE module by applying pressure to the feet. Unlike other studies on shoe-mounted piezoelectric elements, this study focused on providing a step-by-step design approach for a real BLE module. First, we showed how to derive the capacitance and charging voltage required for the BLE power supply capacitor when the necessary parameters are clear in the BLE data sheet. We also showed a measurement approach for the same purpose. Second, we showed an approach to derive the equivalent circuit of the piezoelectric element to enable the batteryless circuit design and simulation of the charging process during walking. Finally, we conducted a verification experiment for a commercial BLE module. The information was successfully transmitted from the BLE module to a smartphone once every 4–5 min of walking when the capacitance of the power supply capacitor is 1620 μF for a charging voltage of 3.8 V, or 940 µF for a charging voltage of 5.5 V. It is also confirmed that the charging time was reduced to one-quarter when four piezoelectric elements were used, compared to the case in which only one piezoelectric element was used. The developed approach will help in the optimization of piezoelectric generator circuits and their performance analysis in the design of batteryless wearable communication modules.

In this study, a normal walking speed of 2 steps per second was adopted. If the walking speed is doubled, the charging time could be reduced by half. This will be confirmed as a future subject in exploring the performance at various walking speeds. Another future subject is the application of the developed batteryless BLE module in the position identification system of elderly wanderers.

## Figures and Tables

**Table 1:** Electrical characteristic of diodes A and B.

Model	Parameter	Condition	Min.	Typ.	Max.	Unit
A (PRE07VS4S)	Forward voltage	$I_{F}=0.2\;A$	-	0.95	1.1	V
	Reverse current	$V_{R}=400\;V$	-	0.01	1	μA
B (RR2L4STE25)	Forward voltage	$I_{F}=2\;A$	-	0.9	1.1	V
	Reverse current	$V_{R}=400\;V$	-	0.01	10	μA

**Table 2:** The time required to boost the voltage to the charging voltage of 3.8 V.

Capacitor	Type of Diode	Experiment	Simulation
200 µF	A	207 s	272 s
	B	250 s	272 s
400 µF	A	494 s	542 s
	B	523 s	543 s

**Table 3:** Walking time required for charging.

Condition		Walking Time [s]	
Charging Voltage [V]	Capacity [µF]	1 Element	4 Elements
3.8	1620	1180	293
5.5	940	-	230

## Data Availability

Data are contained within the article.
